# Amino Acid Changes in Disease-Associated Variants Differ Radically from Variants Observed in the 1000 Genomes Project Dataset

**DOI:** 10.1371/journal.pcbi.1003382

**Published:** 2013-12-12

**Authors:** Tjaart A. P. de Beer, Roman A. Laskowski, Sarah L. Parks, Botond Sipos, Nick Goldman, Janet M. Thornton

**Affiliations:** European Molecular Biology Laboratory, European Bioinformatics Institute (EMBL-EBI), Wellcome Trust Genomes Campus, Cambridge, Cambridgeshire, United Kingdom; Rutgers University, United States of America

## Abstract

The 1000 Genomes Project data provides a natural background dataset for amino acid germline mutations in humans. Since the direction of mutation is known, the amino acid exchange matrix generated from the observed nucleotide variants is asymmetric and the mutabilities of the different amino acids are very different. These differences predominantly reflect preferences for nucleotide mutations in the DNA (especially the high mutation rate of the CpG dinucleotide, which makes arginine mutability very much higher than other amino acids) rather than selection imposed by protein structure constraints, although there is evidence for the latter as well. The variants occur predominantly on the surface of proteins (82%), with a slight preference for sites which are more exposed and less well conserved than random. Mutations to functional residues occur about half as often as expected by chance. The disease-associated amino acid variant distributions in OMIM are radically different from those expected on the basis of the 1000 Genomes dataset. The disease-associated variants preferentially occur in more conserved sites, compared to 1000 Genomes mutations. Many of the amino acid exchange profiles appear to exhibit an anti-correlation, with common exchanges in one dataset being rare in the other. Disease-associated variants exhibit more extreme differences in amino acid size and hydrophobicity. More modelling of the mutational processes at the nucleotide level is needed, but these observations should contribute to an improved prediction of the effects of specific variants in humans.

## Author Summary

In this paper we compare the differences between ‘natural’ and disease-associated amino acid variants at both sequence as well as structural levels. We used data from the 1000 Genomes Project (1 kG), the OMIM database and UniProtKB Humsavar. The results highlight the complex interplay of features from the level of the DNA up to protein sequence and structure. The codon CpG dinucleotide content plays a large role in determining which amino acids mutate. This in turn affects the mutability of amino acids and a clear difference was seen between non-disease and disease variants where amino acids that are naturally very mutable show the opposite trend in the disease-associated data. The current results show evidence for some selection, mainly in that the variants occur slightly more often on the surface of the protein and are much less likely to be annotated as functional than expected by chance. However we should note that even the best definition of functional, taken from structural data, is limited. Even with these caveats, it is clear that the 1 kG variants eschew functional residues as defined here, a trend which is surprisingly even stronger in the OMIM data.

## Introduction

With the release of the 1000 Genomes Project (1 kG) data [1], it has become feasible to study human protein variation on a large scale. The main aim of the 1 kG project was to discover and characterize at least 95% of human DNA variants (with a frequency of occurrence of >1%) found in multiple human populations across the world. Five main populations were sampled with ancestry in Europe, West Africa, the Americas, East Asia and South Asia. The project has provided a rich set of synonymous (sSNPs) and non-synonymous (nsSNPs) variants for 1092 individuals from diverse populations. It is estimated from the 1 kG data that each individual will, on average, differ from the reference human genome sequence at 10,000–12,000 synonymous sites in addition to 10,000–11,000 non-synonymous sites [1]. As these nsSNPs change the amino acid sequence of the protein, the changes have the potential to affect the structure and function of the corresponding proteins. The 1000 Genomes Project data set is valuable in that it is large and not derived from a disease cohort but rather seeks to capture variants found in a disparate set of healthy individuals. This can be used to characterise differences on average between disease-associated and benign mutations (or at least mutations not known to be associated with disease) as well as exploring their structural characteristics and preferences. The reports from the 1000 Genomes Consortium [1], [2] have focused on genome and nucleotide variation, and other papers consider mutations in association with a specific disease (e.g. cancer) [3].

Various databases such as the Online database of Mendelian Inheritance in Man (OMIM, [4]), the UniProtKB human polymorphism set (Humsavar, [5]) and the Human Gene Mutation Database (HGMD, [6]) collect information on inherited diseases associated with variants. The Humsavar database contains disease-associated variants from the literature and OMIM. OMIM currently contains information on approximately 10,200 nsSNPs associated with diseases (December 2011) and Humsavar about 23,500 disease-associated nsSNPs. Most of the phenotypical effects and their molecular origins are not well established, so predicting the functional effect of a single amino acid variant is of great medical interest. The main methods assume that mutations in highly conserved residues cause disease and thus, by using alignments to homologous sequences and residue similarity, the severity of the variant can be gauged. More advanced methods include information derived from protein structures (such as solvent accessibility, free energy changes, environment specific substitution tables and functional annotations) to improve the accuracy (see review by [7]). The advantage of using a 3D approach for prediction is that the consequence and characteristics of the variant can be studied in its specific environment in the protein. This provides a level of information beyond a sequence or a sequence alignment [8]. If there are ligands present, the interaction between the mutated amino acid and the ligand can be studied. This has been successfully applied to various individual proteins on a case-by-case basis [9], [10]. In total over 30 different programs to predict the effects of these variants have been published, including Condel [11], SNAP [12], SDM [13], PolyPhen [14], VEP [15], SIFT [16], [17] and SNP&GO [18]. Most of these algorithms can only predict whether a specific variant will be neutral or deleterious for the protein with various degrees of accuracy, although measuring accuracy is challenging in the absence of a good benchmark.

To allow the accurate prediction of functional effects of SNPs, we need a thorough understanding of why amino acids mutate in humans. Various groups have worked on the effect of the mutations and numerous studies have been done on small specific sets of proteins [8], [19]–[22]. Blundell and co-workers have found that the local environment around an amino acid plays a large role in the effect that selection has on a mutation in a specific position [21]. This has led to the development of environment specific substitution matrices [23], [24] that incorporate structural constraints. Subramanian and Kumar [25] did a detailed analysis on a set of 8,627 disease-associated mutations and found that disease-associated mutations tend to occur on inter-species conserved residues. The common factor between these studies is that they try to understand the effect that selection and structural constraints have on disease vs non-disease states in selected sets of proteins. Very few studies have tried to unravel the underlying cause for mutation patterns seen in human proteins. With this work we aim to elucidate why certain amino acids mutate more and try to understand the underlying mechanisms present in the mutation process. We gather the data for all the amino acid mutations found in the 1000 Genomes Project to characterise their sequence and structural properties, providing a benchmark background against which to compare the disease-associated nsSNPs in OMIM and Humsavar.

## Results

The 1000 Genomes Project data were queried to retrieve all the nsSNPs, which were filtered to include only those that occurred in a single population (see methods). This ensures that only the more recent mutational events in human evolution are included and simplifies counting. In addition variants at a single site were only counted once even if they occur in multiple individuals, since such clusters are assumed to represent a single variation event that has been inherited in the other individuals. For 3D analysis only human proteins, for which complete structures are available, were included to ensure accurate analysis of 3D features. For solvent accessibility calculations, a monomer subset was also generated to avoid problems with uncertain multimeric states and validate our findings on the larger dataset. Homology models based on close relatives were used to extend the data set and see if the trends observed in the experimental structures were preserved. Table 1 summarizes the five data sets created and used in this study.

**Table 1:** The different datasets constructed and used in this study and their composition.

Data set	Protein chains	nsSNPs	Description
1 kG	19,058	106,311	A data set containing all the 1 kG variants filtered by population.
OMIM	19,058	10,151	A protein sequence based set containing OMIM variants for all reviewed UniProt human proteins.
Humsavar	19,058	23,846	A set based on human disease polymorphisms from UniProt.
3D	2,139	10,628	A protein 3D structure based set consisting of 1 kG variants for proteins that have a complete structure in the PDB.
Monomer	325	1,461	A subset of the 3D set containing only proteins identified as being monomeric.
Model	2,630	13,037	A set based on human ModBase homology models where sequence coverage and identity are between 90–100%.

### The amino acid exchange matrix derived from the 1000 Genomes Project dataset

**Figure 1:**
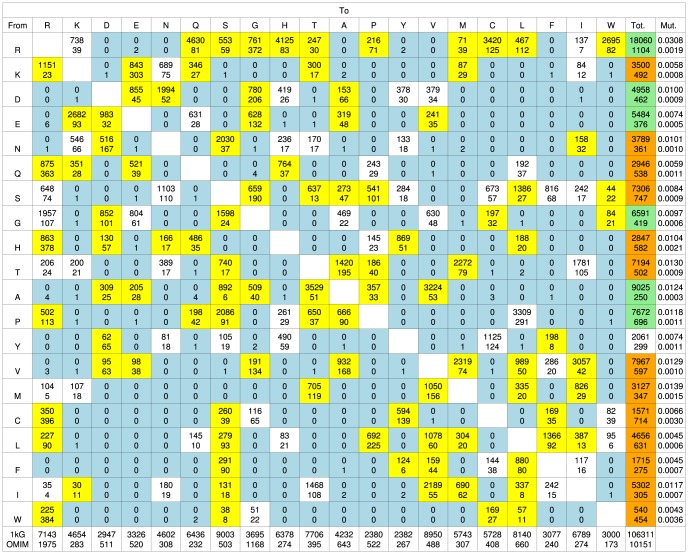
The amino acid exchanges observed in human protein variants. The 1*. Amino acids are arranged by 1 letter code according to increasing hydrophobicity (least hydrophobic is left and most hydrophobic is right) using the Fauchère and Pliska scale [58]. Yellow blocks indicate mutations where there are statistically significant differences between 1 kG and OMIM. Blue blocks indicate where no mutations were present in the 1 kG data set. White blocks show where there are no statistically significant differences. Green blocks show where there are proportionally more 1 kG mutations compared to OMIM. Orange blocks show where there are proportionally more OMIM mutations than 1 kG. The mutability scores (see methods) for the 1 kG and OMIM sets are shown in the last column. ^*^Note that these matrices are fundamentally different. The 1 kG data set gathers all the observed mutations in the 1 kG project, counting each only once; the OMIM data set combines information gathered from potentially many individuals but filtered to identify those mutations associated with a disease.

**Figure 2:**
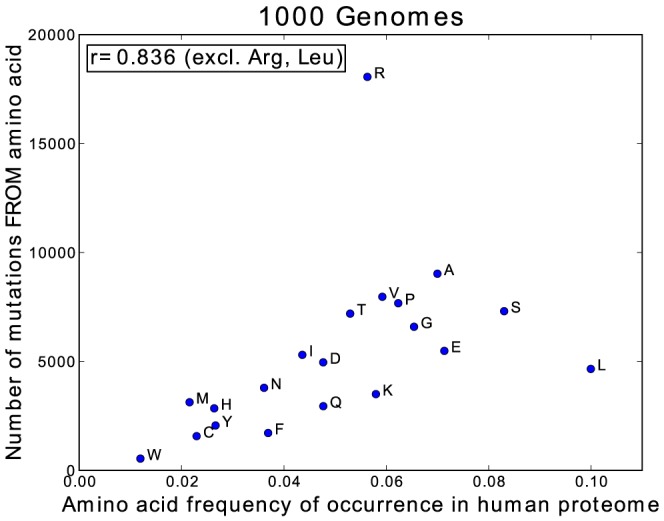
Comparison of the number of mutating residues vs the amino acid frequency of occurrence.

Figure 1 shows the amino acid exchange matrix generated from the ∼106,000 nsSNPs found in the 1 kG data. Amino acid mutations requiring two or three base changes are not defined in this dataset due to technical reasons. The 1 kG matrix exhibits several interesting features, most of which reflect the genetic code and the differential mutability of various codons. All possible single base changes are observed. The matrix is not symmetrical as a result of the differences in frequency of occurrence of amino acids as well as differences in their mutabilities [26], [27]. As expected there is a strong correlation (r = 0.786) between the frequency of occurrence of amino acids in the human proteome and the number of associated codons. Figure 2 shows that, excluding Arg and Leu which are extreme outliers, there is a strong trend for amino acids with a higher frequency of occurrence to have more mutations (r = 0.836). Taken together this leads to a relatively strong correlation (r = 0.741) between the number of codons and the number of mutations. In contrast, the frequency of the gained amino acids, resulting from the mutation, shows little correlation between frequency of occurrence and number of mutations (r = 0.349).

### Amino acid mutabilities

**Figure 3:**
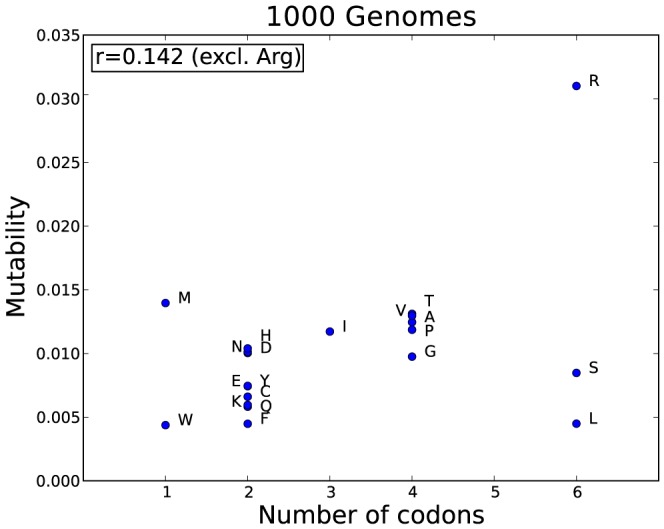
Amino acid mutability vs the number of codons in the 1 kG data.

**Figure 4:**
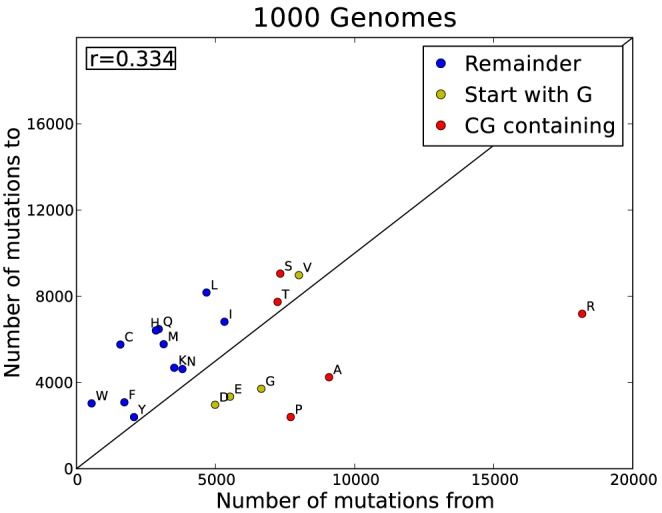
A visual representation of the asymmetry of the 1 kG data. The plot shows the difference between how often an amino acid mutates vs how often it is mutated to. These are raw counts and also reflect the frequency of occurrence. Each amino acid is coloured according to CpG content. Red: a CpG dinucleotide occurs in its codons; yellow: if one of its codons start with a G (with a C possibly preceding it); blue: no CpG in its codons. The black line indicates the diagonal where ‘mutations to’ equals ‘mutations from’.

The mutabilities of the amino acids (see methods) in the 1 kG dataset are shown in the last column of Figure 1. Arg (0.031) is the most mutable, whilst the more chemically complex amino acids, Trp (0.004) and Phe (0.005) have the lowest mutabilities. There is no correlation in the 1000 Genomes data between mutability and frequency of occurrence (r = −0.003 excluding Arg) nor between mutability and the number of codons (Figure 3). It is well known that CpG dinucleotides in DNA tend to mutate at rates 10–50 times higher than other dinucleotides [28], [29] and thus amino acids with a CpG present in their codons will mutate with a higher probability (see Figure 4). Four out of the six codons for Arg include CpG sequences, and Arg mutates more frequently than any other residue, with a mutability (0.031) which is over twice as high as its nearest rival. This high mutability also reflects the fact that the CpG in the Arg codons occur in the non-wobble positions so nucleotide mutations give rise to non-synonymous SNPs. In contrast Leu which also has six codons, none of which contain CpG, has a low mutability (0.005) and mutates six times less frequently than Arg. However the correlation with CpG is far from perfect and other factors must have an effect. For example, Met, which has only one codon with no CpG dinucleotide, is the second most mutable amino acid (0.014).

Figure 4 shows the clear pattern of amino acid gain and loss in the human proteome. Jordan [26] and Zuckerkandl [30] long since identified that Cys, Met, His, Ser and Phe are being accrued significantly in the human proteome. Our data confirm a net gain of these five amino acids, and Val, Asn, Ile and Thr were also confirmed as weak gainers. Jordan and co-workers also identified strong losers and our data again confirm that Pro, Ala, Gly and Glu are strong losers. Lys was identified as a weak loser but our larger dataset suggests that lysine should be considered a weak gainer in humans. Arg is the strongest loser in the human genome (similar to the human set in [26] but not other considered species).

We calculated the mutability for every amino acid on a population specific basis. None of the populations showed a different pattern of amino acid mutabilities, compared to the overall trend with correlation coefficients equal to 1.0 (Figure S1). Using the individual amino acid mutabilites, we looked at aggregate protein mutability differences by adding up the individual mutabilities for every amino acid in each protein in the data set and normalising by protein length. This was compared to the aggregate mutabilities of proteins involved in disease as classified by OMIM and Humsavar. The average score for disease-associated proteins was 0.0103 and for non-disease proteins 0.0102 with a median of 0.01022 (*σ* = 0.0006) and 0.01018 (*σ* = 0.0005), respectively, indicating that protein aggregate mutability has no bearing on disease-association (Figure S2).

### The effects of physicochemical characteristics of the amino acids on their mutability

As well as constraints on the mutational process at the DNA level, the consequence of a variant on the protein structure and function will also have an impact on the number of observed mutations. If a variant interferes with the structure and function of a protein and that protein is essential, then this variant is less likely to be seen. However comparison of mutability with the size and hydrophobicity of the amino acid shows very little correlation in the 1 kG dataset. There is a moderate anti-correlation between higher mutability and size (r = −0.474), with the smaller amino acids mutating more frequently, but no correlation at all between mutability and hydrophobicity (r = −0.082) although the large hydrophobic amino acids (Leu, Phe and Trp) have the lowest mutability scores. Trp has the fewest mutations (544, even though all SNPs in Trp codons result in a change of amino acid) and also the lowest mutability score (0.004) together with Phe. In addition to their complexity and low abundance, Phe and Trp often occur in specialized roles such as the interior of proteins, *π*-π stacking or ring interactions and this might add to their low mutability. The mutability of Cys is also low, perhaps reflecting its role in disulphide bridges, which help to stabilise extracellular proteins.

### The structural properties of 1000 Genomes variants

To investigate the structural characteristics of these variants, three sets of protein structures were compiled, namely the 3D set, the monomer set and the model set (Table 1). The 3D and monomer set were constructed from data in the PDB (see methods) while the model set and the subsequent variant modelling was created and performed using Modbase [31] and Modeller [32], built into an in-house homology modelling pipeline. The 3D set contains 2,139 protein chains. A total of 10,628 1 kG nsSNPs were found in these chains, of which protein models, based on the known structures of human proteins could be built for 5,524. The monomer set contains 325 protein chains identified as monomers and a total of 1,461 1 kG nsSNPs were found, of which 897 could be modelled. The model set, including models based on homologues from the PDB, contained 2,630 protein chains and 12,432 out of 13,037 nsSNPs could be modelled. For the Humsavar set we found 5,592 nsSNPs of which 3,942 could be modelled.

**Figure 5:**
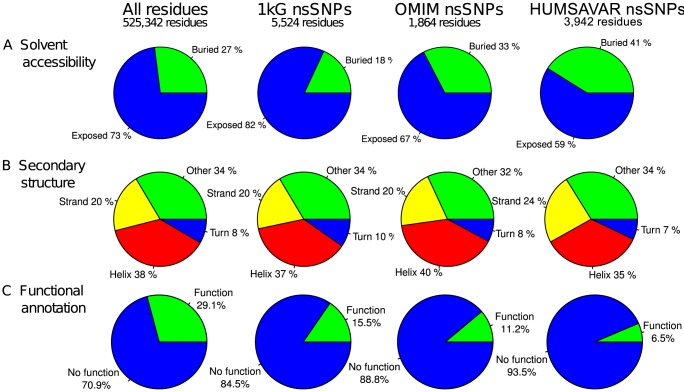
Site properties for all residues, 1 kG nsSNPs, OMIM nsSNPs and Humsavar nsSNPs in the structure 3D set. (A) the solvent accessibility for the variants in the four datasets, (B) the secondary structure in which each of the variants occurs, (C) the functional annotation of every variant in the four datasets.

Figure 5A shows a comparison of the solvent accessibility distribution for all residues compared to that for the variants. On average the variants in the 1 kG are slightly more exposed. An analysis of the solvent exposed residues found that, for the most accurate monomer set, 79% of nsSNPs are solvent exposed compared to 73% of all residues (p = 0.001). For the structures in the model set, 81.9% of nsSNPs were solvent exposed. For all three datasets, the 1 kG variants have a slight preference to occur on the surface of proteins compared to all residues. Figure 5B shows that there were no appreciable differences in secondary structure preferences between variants and other residues.

### Do natural mutations occur in functionally annotated residues?

Functional annotation for each human protein was derived using SAS (Sequence Annotated by Structure, [33]). Table 2 shows the different functional annotations for each set. The vast majority of functional annotations identified, make contacts to ligands (using PDBsum data, [34]) or site interactions in the proteins (as defined in the PDB). Only 15.5% of the mutations (1,648 of 10,628) in the 3D set were annotated with a function compared to 29.1% of all residues in the set of human structures (Figure 5C). These data show that the observed mutations in the 1000 Genomes occur less frequently in the functionally annotated residues compared to all residues.

**Table 2:** The various functions assigned to nsSNPs in each set.

Set	Site	Ligand	Site/ligand overlap	Metal	Catalytic	Overall (non-redundant)
3D	1,414	1,432	1,220	334	17	1,648 (15.5%)
Monomer	281	273	245	83	4	312 (21.4%)
OMIM	163	184	147	17	17	209 (2.1%)
Humsavar	305	285	252	58	41	355 (51.2%)
Models	1,538	1,443	1,304	376	36	1,676 (12.9%)

‘Site’ refers to residue specific annotations made by depositors of PDB structures, ‘Ligand’ refers to residues involved in binding a ligand, ‘Metal’ refers to residues coordinating with metals and ‘Catalytic’ to residues involved in the catalytic activity of the protein. The % of non-redundant assigned residues that are ‘functional’ is also shown.

### Residue conservation

**Figure 6:**
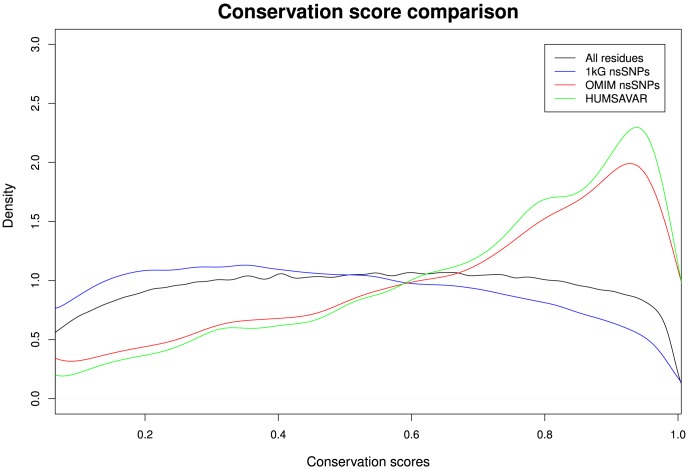
Comparison of the conservation scores in the four sets used. The density distribution of residue conservation scores for all the amino acid positions in UniProt (9,532,474 residues, black), 1 kG (185,428 residues, blue), OMIM (8,099 residues, red) and Humsavar (21,446 residues, green). The conservation scores range from 0 for non-conserved residues to 1 for highly conserved residues.

Residue conservation scores, defined as the variation of the residues at a given site in the protein across multiple species, were obtained for all sites in the human proteome (where sufficient data are available) from the Evolutionary Trace server [35]. These scores are distributed across the whole range of conservation (Figure 6) with a mean score of 0.48. The scores for all the sites with mutations in the 1000 Genomes data show a slightly different distribution from all residues, with a small but significant shift (*p*<2.2×10^−16^) towards the less conserved sites and a reduced mean conservation score of 0.43. Clearly natural variation occurs across all conservation levels and is not limited to non-conserved residues.

### Amino acid exchange characteristics in 1000 Genome data

**Figure 7:**
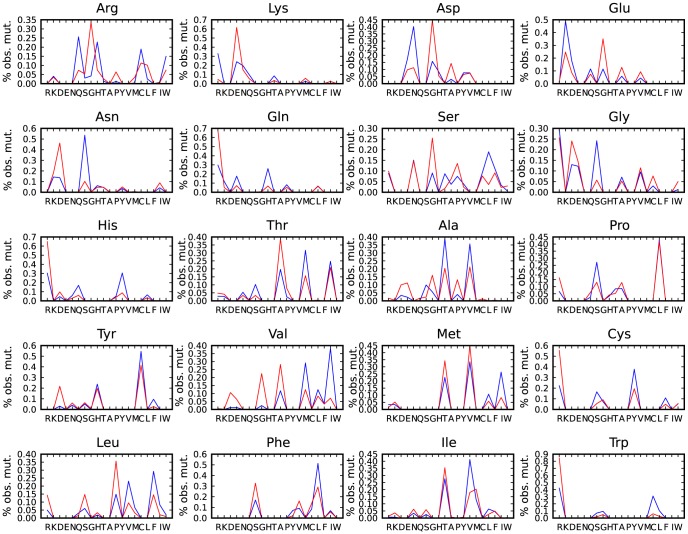
Comparison of the differences in observed mutations in the various sets. Comparison of the differences in the % of observed mutations in the 1 kG (blue) and OMIM (red) sets for one amino acid mutating to all others e.g. proportionally, more mutations from Lys to Glu are recorded in OMIM than in the 1 kG set. Each plot shows the results of mutation from a specific amino acid (e.g. Arg at top left) to every other amino acid.

**Figure 8:**
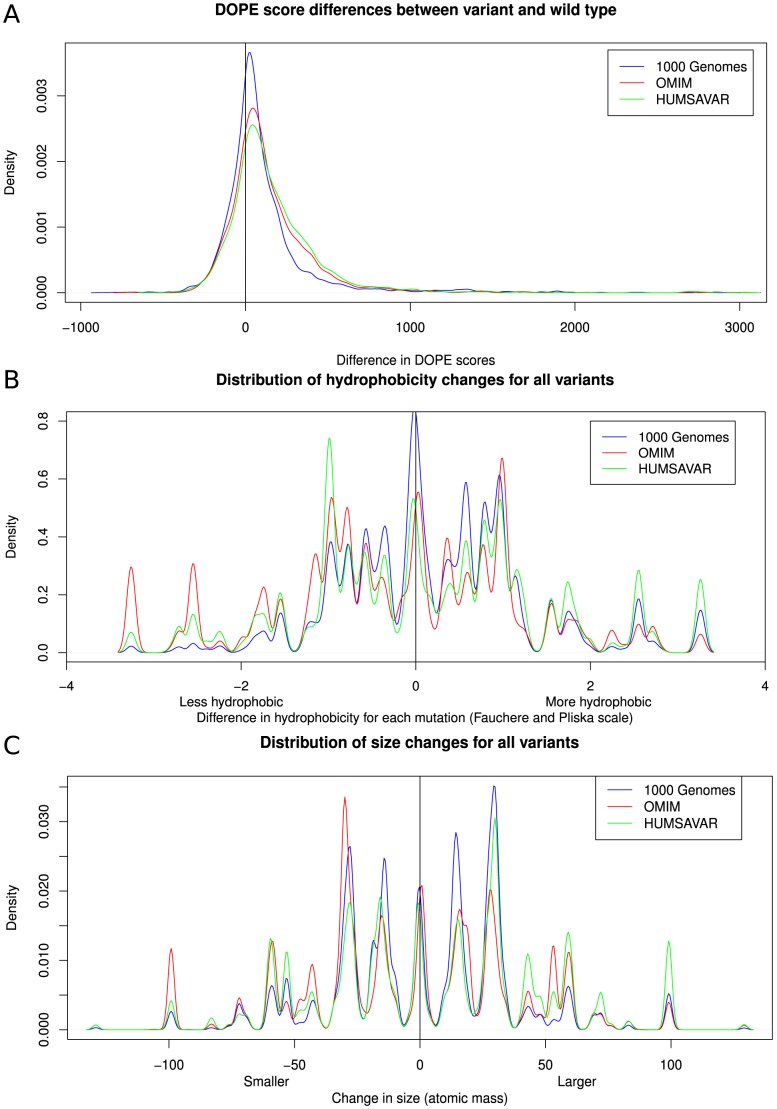
Comparison between the physicochemical properties of the wildtype and the mutant models for each of the data sets. Plots showing the differences between (A) Modeller DOPE scores for the wild type and mutant model (based on 3D, 10,628 mutations, and Humsavar sets, 21,446 residues), (B) changes in hydrophobicity between wild type and mutant in both sets and (C) changes in size between wild type and mutation in both sets.

For each amino acid the mutation profile can be calculated showing the preference for specific X = >Y mutations in the 1000 Genomes data. These profiles, given for all the amino acids in Figure 7, show that there are striking differences in frequency of occurrence for the different exchanges. For example, in the 1 kG set Arg shows a strong preference to mutate to Gln and His, whilst mutations to Ser, Gly and Pro are much less frequent. All the amino acids show these differential exchange rates. Figure 8A shows the distribution of changes in energy of the whole protein caused by each mutation, evaluated as the statistical potential energy DOPE score (Discrete Optimised Protein Energy) in Modeller. 68.1% of the 1 kG variants increase the DOPE score (i.e. make the protein less stable). This implies that most natural variants decrease the stability of the protein, albeit by a very small amount. The distribution of changes in size and hydrophobicity for all observed mutations (Figure 8B and 8C) show that 59.4% of mutations increase the hydrophobicity of the amino acid and 52.4% of mutations increase the size. Over 84% of variants change their size by less than 50 Da. 72% of variants change their hydrophobicity by less than 1 unit. Extreme changes are rare. At this stage these observations provide empirical expectation rates for amino acid exchanges in humans and result from the genetic code, the nucleotide exchange rates and also some selection at the protein level. However without a good random model it is difficult to be confident about the importance of the different contributions to such variation.

### Comparison of 1000 Genome variants with those predicted by the PAM and WAG mutation matrices

**Figure 9:**
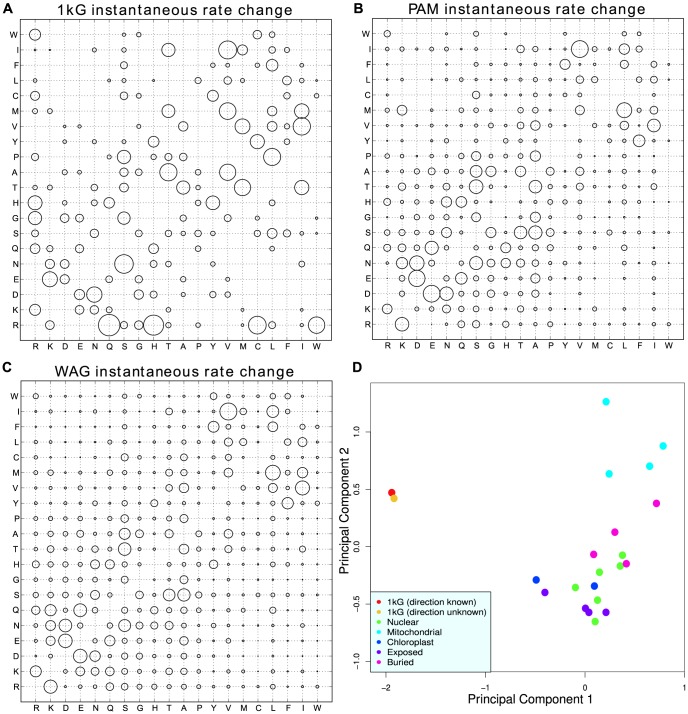
Bubble plots comparing the relative differences between the instantaneous rate change matrices of the data sets. (A) 1 kG data, (B) PAM matrix and (C) WAG matrix. (D) A PCA (first two components) plot showing the separation of the 1 kG matrices from other matrices. Matrices included are 1 kG (with and without assuming direction), nuclear (WAG, JTT, LG, PAM, tm126, PCMA), mitochondrial (mtREV24, mtMam, mtArt, mtZoa), chloroplast (cpREV, cpREV64), exposed (alpha helix, beta sheet, coil, turn) and buried (alpha helix, beta sheet, coil, turn). Principal components one and two represent 34% and 20% of the variance, respectively. All other principal components represent 9% or less of the variance each. Amino acids are arranged according to increasing hydrophobicity.

The 1 kG counts matrix is a snapshot of mutations that have occurred in humans in a short period of time. To understand this process the count matrix can be converted into an instantaneous rate matrix describing the rates of change of each amino acid in humans in a time-independent manner [36]. Instantaneous rate matrices have previously been built from a wide selection of protein alignments across many species including nuclear proteins, mitochondrial proteins, chloroplast proteins, buried protein domains and exposed protein domains. PCA can be used to compare these inter-species matrices with the 1 kG intra-species matrix (Figure 9A–C). The 1 kG matrix was built using data where the direction of the mutations is known whereas all other matrices were calculated assuming direction is unknown. This was compared to the WAG [37] and PAM matrix [38]. To check that any differences between the 1 kG matrix and the other matrices are not caused by using direction, a directionless matrix has also been included in the plot (Figure 9D). In this plot, principal component one clearly separates the 1 kG matrices, which are placed very close together, from all of the previously calculated matrices. Principal component two then spreads matrices out based on whether the alignments used to build them are made up mainly of exposed or buried domains, with the mitochondrial matrices at the one extreme built from nearly all membrane proteins, and matrices built from only exposed regions of proteins at the other.

**Figure 10:**
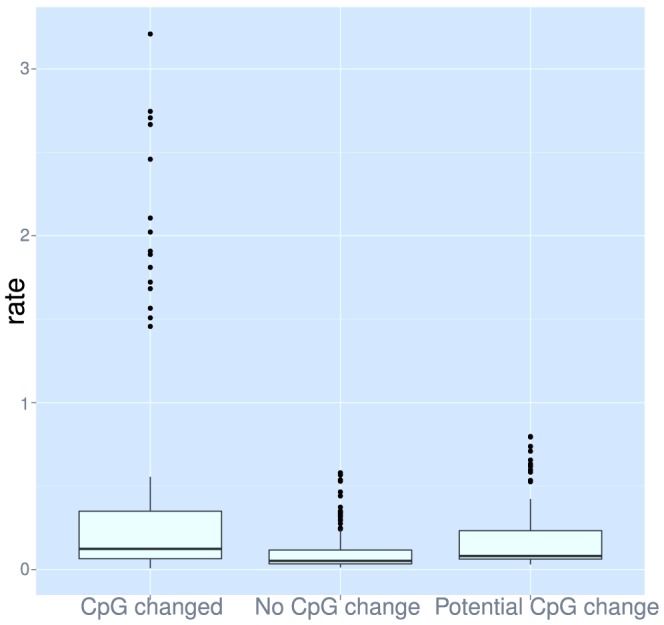
Dependence of mutation rates on the change in CpG status. Rates of change from codons were calculated similarly to the amino acid rate matrix [36], but on a 61 by 61 codon matrix.

A difference between the intra-species data and the inter-species matrices is the amount of selection which has occurred. Due to the time-scale for the 1 kG data and the relatively weak selection in human populations [39], [40] the only mutations which are not observed are lethal mutations. This means that there should be a limited effect of selection on the 1 kG matrix. By using no allele frequency cutoff for the minor alleles when building the count matrix, we gather the maximum amount of information about the mutation process. The counts are necessarily shaped by mutation and selection but will mostly reflect the mutation process. The inter-species matrices (e.g. PAM and WAG in Figure 9B,C) on the other hand are subject to selection pressures. This could explain why the 1 kG matrix is so different from the other matrices. One clear factor is CpG hypermutability: for example, changes from Arg, an amino acid with four of six codons containing a CpG, have a very high rate in the 1 kG data, and not in WAG (Figure 9A,B). In fact only codons containing a CpG have high rates overall (Figure 10). The most plausible explanation is that these CpG mutations are occurring at a very high rate and then are selected out so that the effect is not seen as strongly when looking across multiple species.

### Comparison between the 1000 Genomes variants and the disease-associated variants

For comparison, we have constructed the amino acid exchange counts matrix for data from the OMIM database and the associated plots for these mutations (Figures 1–8). Disease variants from the UniProtKB/Swiss-Prot Human polymorphisms and disease mutations index (Humsavar) were also included with plots available in the supplement (Figures S3, S4, S5). Our focus however is on the OMIM set. In contrast to the 1 kG data, various double and triple base mutations are observed in the OMIM set, however the three triple base changes (Phe-Lys, Met-Tyr and Trp-Ile) were checked back to the publications and all were found to be errors either in the paper or in OMIM and were removed. 82 two base changes were found in OMIM and a few (10%) randomly selected changes were manually checked with no errors found. Clearly the OMIM data are radically different from the 1000 Genome data, in that they are all independent observations of variable confidence and manually determined by individual scientists. They only represent a small fraction of disease-associated nsSNPs and the number of mutations (∼10,000), is approximately ten times smaller than the number of 1000 Genomes mutations. The normalised OMIM counts that differ from the 1 kG dataset are coloured in Figure 1. Considering just the residue type, if we exclude Arg, the overall correlation between the normalised frequencies of occurrence of the mutated residues in the two datasets is only 0.14 and between 1 kG and Humsavar it is 0.48. If we compare all 148 observed X = >Y frequencies, the correlation between 1 kG and OMIM is 0.51 and 1 kG and Humsavar is 0.79.

Previous studies have found that mutations from Arg and Gly are the major contributors to human genetic disease and have been shown to make up about 30% of the mutations involved in disease [41]. In this updated and much expanded set, variants from Arg and Gly only make up 15% of the disease causing mutations. However mutations to Arg are still the biggest contributor to genetic disease with ∼19.4% of all mutations.

**Figure 11:**
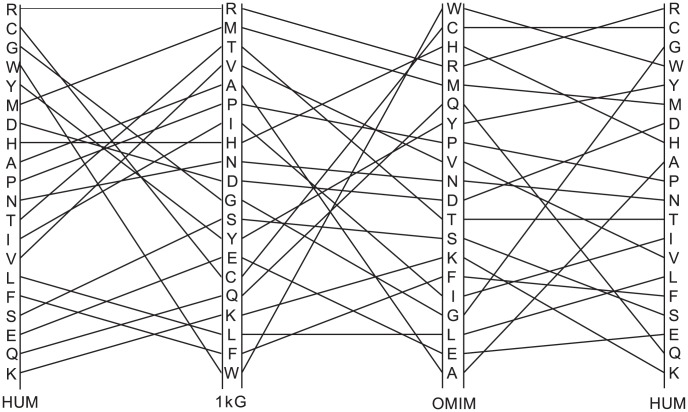
Amino acid mutability rank order plot comparing the mutability scores for 1 kG, OMIM and Humsavar residues. The most mutable amino acids are at the top. Correlation coefficients for 1 kG vs OMIM, 1 kG vs Humsavar and OMIM vs Humsavar are 0.09, 0.17 and 0.51, respectively.

Figure 11 shows a rank order comparison between the frequency of occurrence of the 1 kG and OMIM variants (r = 0.09) as well as between 1 kG and Humsavar (r = 0.31) and Humsavar and OMIM (r = 0.51), normalised for amino acid occurrence. Unlike for the 1 kG data, the disease-associated variants show moderate inverse correlations between their frequency and the frequency of occurrence of the residue type (r = −0.67) implying that, at least for OMIM, the mutations to the rarer amino acids (with fewer codons) are more likely to be associated with disease. As with the 1 kG data there is no strong correlation between a residue type being associated with a disease in the OMIM data and the number of codons. For hydrophobicity and size, the disease associated variants show the opposite trend to the 1 kG dataset with a moderate correlation between lower frequency and smaller size (r = 0.528, excluding Cys and Trp) but no correlation between frequency and hydrophobicity (r = 0.289). It is interesting to note that the least mutable amino acid in the 1 kG data (Trp) turns out to be the residue whose mutation is most likely to result in disease in the OMIM variants and is highly ranked in the Humsavar set. Trp, the largest amino acid, often occurs in specialized roles in proteins as does Cys, the second most frequent variant residue type in OMIM. Amino acids with a lower frequency of occurrence tend to be the more complex amino acids and are frequently found in specialized roles. Mutating them will result in the possible loss or alteration of protein function, hence the over-representation in OMIM and Humsavar. In a number of cases the OMIM and 1 kG variant preferences appear to behave in an opposite way from one another e.g. in Figure 7 Arg most frequently mutates to Gln in the 1000 Genomes and a variantion to Gly is much less common, whilst Arg to Gly is the most common variant in the OMIM dataset and a variation to Gln is rare.

We observe a reasonable correlation between the OMIM and Humsavar mutabilities (r = 0.51), but some amino acids appear to behave completely differently in the two datasets. Gly and Ala are much more frequently mutated in the Humsavar set than in OMIM, whilst Gln, Lys and His have mutabilities in the Humsavar set similar to those observed in the 1 kG dataset and much smaller than those in OMIM. This may reflect the larger Humsavar dataset (but this seems unlikely since Gly and Ala are quite common amind acids), so these specific discrepancies may rather reflect the origins of mutations in the two separate datasets.

### Structural properties of disease-associated nsSNPs

The disease-associated OMIM variants show a slight preference for buried sites (33%) compared to all residues (27%) in the human proteome (Figure 5A) is even stronger in the Humsavar data (41%). This contrasts with the ‘natural’ variants of the 1 kG data, which show a decreased preference (18%) for the interior. Our work broadly agrees with a smaller study done by Gong and Blundell [21] that showed 60–65% of disease associated nsSNPs are solvent exposed. We found an almost identical distribution of OMIM and Humsavar variants compared to all residues and the 1 kG variants between the different secondary structures (Figure 5B).

Figure 8A shows the differences in the DOPE scores [42] calculated for each variant during the structural modelling process for the 1 kG, OMIM and Humsavar datasets. The distribution for the disease-associated variants is shifted towards larger positive energies in both datasets, indicating that the variants destabilize the protein slightly more than the non-disease variants. In contrast to the 1 kG data, OMIM mutations are more likely to increase polarity (54%) and more likely to decrease size (51.6%, Figure 8B,C). The two datasets show some detailed differences in size and hydrophobicity changes. The Humsavar variants less frequently reduce size or decrease hydrophobicity compared to OMIM mutations.

### Functional annotations

In the OMIM set, 11.2% (209 of 1,864) of the modelled mutations were annotated with a function (Figure 5C and methods). This is less than the distribution for all residues (29.1%) and that seen for the 1 kG variants (15.5%). For the Humsavar data this drops to only 6.5%. This is a surprising finding, which needs further validation. It implies that most disease-associated mutations do not have a direct effect on the proteins' catalytic or binding sites but instead act through other, unannotated residues such as those which affect overall structure and stability or are involved in as yet unidentified protein-protein interfaces.

### Conservation

There is a clear difference in the conservation score distribution between natural variants and the OMIM and Humsavar variants (Figure 6). The natural variants occur across the entire range of conservation but the OMIM and Humsavar variants show a peak in the more conserved residues. This is consistent with the idea that mutations in conserved residues often lead to disease.

## Discussion

The results presented herein are subject to a few caveats, the most serious being related to the limited and possibly biased disease-associated data in OMIM. There are only ∼10,000 variants in our OMIM set and these have variable experimental validation, and may indeed be biased according to scientists' preconceptions that such mutations should correspond to the residues that are most conserved and the amino acid exchanges that generate the largest changes in physicochemical characteristics. The Humsavar set has over 23,000 disease variants, however the requirements for inclusion are based on an annotation of ‘involvement in disease’. This annotation is derived from either OMIM annotations or associations found in literature during curation of the SwissProt data. Notwithstanding, the OMIM dataset is one of the best available at the present time, although the coming years will see major expansion and hopefully improvements in such data. The results highlight the complex interplay of features from the level of the DNA up to protein sequence and structure. The codon CpG dinucleotide content plays a large role in determining which amino acids mutate. This in turn affects the mutability of amino acids and a clear difference was seen between non-disease and disease variants where amino acids that are naturally very mutable, show the opposite trend in the disease-associated data.

The data for the 1000 Genomes provides a new experimental baseline against which amino acid profiles may be compared. Although there might be sequencing biases due to the DNA sequencing techologies used [43], every effort has been made by the 1000 Genomes consortium to correct for this. They estimate that using consensus calling on data produced by multiple platforms results in an error rate of 1–4%, thus having a small but negligible impact on our results. The current results show evidence for some protein selection, mainly in that the variants occur slightly more often on the surface of the protein and are much less likely to be annotated as functional than expected by chance. However, we should note that even the best definition of functional, taken from structural data, is limited. At one level, the definition is rather broad. For example, all residues in contact with a ligand are described as functional, but this is a major underestimate since many cognate ligands are not present in the crystal structures and similarly protein-protein interactions are rarely captured. In addition there are still relatively few complete structures for human proteins, which makes analysis of the effects of variants more difficult.

Even with these caveats, it is clear that the 1 kG variants eschew functional residues as defined here, a trend which is surprisingly even stronger in the OMIM and Humsavar data. The preference for OMIM mutations to be more buried and less functional supports the suggestion that these variants predominantly affect the structure and stability of the protein [4]. This is a similar result to that found by Sunyaev and co-workers [44] on a much smaller set. They found that 35% of disease variants were buried and a more detailed analysis found that ∼70% of the variants are located in structurally and functionally important regions. Therefore these disease-associated mutations may well target residues that are remote from the active site, which modulate rather than obliterate the function of the protein. For example, for an enzyme, the primary catalytic residues are rarely targeted, but the ‘secondary’ residues in the interior (which affect stability) or on the surface, which may affect protein-protein interactions, could modulate function. However, the higher than average conservation scores for OMIM and Humsavar sites suggest that these disease-associated residues, although not defined as ‘functional’, are still important for the organism. This needs further investigation, with particular attention to how ‘functional’ residues are defined and whether we can improve on this definition.

Bringing together all the above observations for disease-associated and natural variants in _∼_1000 humans, we observe that the mutability of amino acids is largely driven by the properties of the DNA and mutational mechanisms, which favour mutations at codons containing a CpG dinucleotide. Therefore mutations to Arg residues are more than twice as common as any other mutation. However there are clearly other factors at play, which determine the frequency of variants, even at the DNA level. Although the disease-associated variants (both OMIM and Humsavar) follow the same pattern as the 1 kG variants (i.e. the same mutations are present in both sets, as dictated by the genetic code), the rank order of amino acids, according to their probability of being disease-associated, is radically different from that expected on the basis of the 1 kG data, with some of the rarer amino acids being shifted to the top of the list.

There is a small but significant impact of the protein structure on amino acid mutability, so that natural variants occur slightly more often in non-conserved regions. 59.4% of variations increase the hydrophobicity of the amino acid and 52.4% increase its size in the natural set, while OMIM variants often result in larger changes in the size and hydrophobicity of the amino acid and are more destabilising on average than 1 kG variants. The Humsavar data supports this idea that disease variants result in more extreme changes. The selection pressures captured in the WAG and PAM matrices ‘purify’ out the ‘natural’ variants, removing variants with large changes in size and hydrophobicity. The amino acids all show distinctive exchange profiles, whereby some exchanges are very common and some very rare, which provides an empirical expectation for any specific exchange in humans.

As the cost of sequencing drops rapidly, many more genomes will be sequenced and experimental validation of disease-causing mutations will improve as a result of more data. Much better codon-based models of evolution will be attainable, allowing in turn a better dissection of the impact of selection at the protein level. The data herein will be used to develop an improved method to predict the effects of individual mutations, to explore cancer-related amino acid mutations, to investigate and compare mutational profiles in different organisms as well as improving codon mutation models for human DNA.

## Methods

### Non-synonymous mutations in humans

UniProt [5] was queried for all reviewed protein sequences belonging to *Homo sapiens*. 19,058 entries were retrieved. The Ensembl transcript ID [45] was obtained for each protein sequence using the mapping provided by UniProt (17,708 UniProt entries were mapped to 40,351 Ensembl transcript IDs). Immunoglobulins and major histocompatibility complex proteins were excluded as they are inherently variable. For every protein, the Ensembl v67 Perl API was used to query the transcript ID in Ensembl for nsSNPs found in the 1 kG data set (as available on 1 August 2012). To reduce the inherent uncertainty involved in determining the ancestral allele, only mutations that occurred in one of the 1000 Genomes described populations were used, with the allele present in all populations considered the ancestral, hence defining the direction of the mutation. This increases the chances that the variant found in the 1 kG data is a mutation away from the ancestral genome. 106,311 mutations were found and this data set, containing the ‘natural’ variants found in the 1 kG project, will be referred to as the 1 kG set.

Residue conservation scores for each residue in every protein sequence were calculated using the Evolutionary Trace server [35]. Conservation scores for 2,274 sequences could not be calculated due to the methodology used by the Evolutionary Trace server that disregards residues in columns of the multiple alignment containing more than 60% gaps and ranked as being non-conserved, as well as residues judged by the algorithm not to have enough information. This process almost certainly preferentially excludes surface residues (where insertions and deletions are most common) but since we are using the conservation distribution for comparisons, this bias is not significant. The UniProt sequences were used to calculate the relative abundance of amino acids in human proteins. A total of about 10.5 million amino acids were counted. For each protein sequence, the OMIM Mutations search tool (http://www.bioinf.org.uk/omim) was queried with the UniProt entry ID to retrieve variants found in OMIM. Only variants for which the correct amino acid position in the protein has been verified, were used for the OMIM data set and will be referred to as the OMIM set. 556 of the OMIM mutations were found in the 1 kG set (0.5%). Although these represent a very small fraction we removed them so that they did not bias the results.

The instantaneous rate change matrices were derived using the DCFreq method [36] and the human proteome frequencies.

### Mutability of amino acids

A mutability score for every amino acid was calculated by taking the total number of mutations for a specific amino acid in the data and dividing by the frequency of occurrence for the specific amino acid in the human genome. The proportional representation of each amino acid in the human proteome is given in supplemental Table S1.

### Statistical validation

We compared the amino acid variant counts in the 1 kG and OMIM data using Fischer's exact test in the R package (R Development Core Team, 2011). Multiple comparison correction was done on the p-values for each amino acid using p.adjust in R with the Benjamini-Hochberg-Yekutieli method [46], [47]. P-values lower than 0.01 were considered statistically significant. For correlation values, r>0.7 and r<−0.7 were considered strong, 0.4<r<0.7 and −0.4>r>−0.7 were considered moderate and 0.3>r>−0.3 weak or no correlation.

### Retrieving human proteins and their structures

The protein structure data set was constructed by first taking all the above mentioned protein sequences and annotating each with their respective Pfam [48] domains. Only proteins for which there were matching entries in the Protein Data Bank (PDB, [49]) were kept. This resulted in a list containing the UniProt identifiers for all known human proteins that have at least one structure in the PDB. For accuracy, the corresponding PDB structures were then filtered to include only X-ray structures. Using the Pfam mapping, only protein structures containing all the protein's Pfam domains were kept. The final list contained 2,139 protein chains and will be referred to as the 3D set.

A set consisting only of human monomeric proteins was also constructed. An algorithm was implemented whereby a protein was classified as being either a multimer or a monomer based on a majority vote. The predictions used were from PISA [50], UniProt, 3DComplex [51], PIQSI [52], PQS-PITA [53]–[55], relevant PubMed abstracts and REMARK 350 records from the PDB structure file. The oligomeric predictions from each of the servers were collected for every protein in the 3D set. Only when the majority of the servers agreed on the most probable oligomeric state of the protein, was it designated as either a multimer or a monomer. The monomeric protein list contained 325 proteins and will be referred to as the monomer set.

Another homology-based set was constructed using the human models in ModBase [31]. Models with 90–100% sequence identity and coverage were used as templates. This set contained 2,630 models and will be referred to as the model set.

### Protein chain annotation

Each protein chain in the 3D, monomer and model sets was annotated with information from various databases and online resources. Information about protein properties such as catalytic residues, metal-binding residues, ligand-binding residues and PROSITE patterns [56] were extracted from PDBsum [34] and additional functional residue annotations were retrieved using SAS (Sequence Annotated by Structure, [33]). The 3D coordinates for each of the proteins in the structure data sets were retrieved from the PDB. To maintain consistency between the PDB and UniProt residue numbering, the SIFTS mapping [57] for each protein chain was used. NACCESS was used to calculate the relative solvent accessibilities for the individual residues in a chain. A cut-off of 5% solvent exposure was used to distinguish between buried and exposed residues.

### Mapping nsSNPs to structures

To investigate the effect a nsSNP might have, each individual nsSNP was mapped to its correct amino acid in the protein structure. For every such nsSNP that could be mapped, a homology model of the protein containing the nsSNP was built using Modeller 9v3 [32] with the original protein structure serving as the template. A maximum of 200 steps of conjugate gradient minimization followed by 200 rounds of molecular dynamics at 300 K (using Modeller) was applied to each variant and its structural context analysed. NACCESS was run on all the variant models to identify changes in solvent accessibility. Comparisons of the Modeller DOPE score (Discrete Optimized Protein Energy, [42]) were made between the nsSNP model and the reference structure to estimate the magnitude of change that a variant might cause. The 1 kG models are available in PDBsum (http://www.ebi.ac.uk/pdbsum/) by looking at the specific PDB code of interest.

## Supporting Information

Figure S1 **Mutabilities of the amino acids for each population. AMR: American admixed, ASN: South East Asian, AFR:African, EUR: European.**
(EPS)

Figure S2 **The distribution of average protein mutabilites for all human proteins (blue) and disease associated proteins (red).**
(EPS)

Figure S3 **The amino acid exchanges observed in human protein variants.** The 1 kG data set is the top row of each cell and Humsvar(SP) the bottom row of each cell*. Amino acids are arranged by 1 letter code according to increasing hydrophobicity (least hydrophobic is left and most hydrophobic is right) using the Fauchère and Pliska scale. Yellow blocks indicate mutations where there are statistically significant differences between 1 kG and Humsavar. Blue blocks indicate where no mutations were present in the 1 kG data set. White blocks show where there are no statistically significant differences. Green blocks show where there are proportionally more 1 kG mutations compared to Humsavar. Orange blocks show where there are proportionally more Humsavar mutations than 1 kG. The mutability scores (see methods) for the 1 kG and Humsavar sets are shown in the last column. *Note that these matrices are fundamentally different. The 1 kG data set gathers all the observed mutations in the 1 kG project, counting each only once; the Humsavar data set combines information gathered from potentially many individuals but filtered to identify those mutations associated with a disease.
(EPS)

Figure S4 **Comparison of the differences in observed mutations in the various sets.** Comparison of the differences in the % of observed mutations in the 1 kG (blue) and Humsavar (red) sets for one amino acid mutating to all others e.g. proportionally, more mutations from Lys to Glu are recorded in Humsavar than in the 1 kG set. Each plot shows the results of mutation from a specific amino acid (e.g. Arg at top left) to every other amino acid.
(EPS)

Figure S5 **Comparison of the differences in observed mutations in the various sets.** Comparison of the differences in the % of observed mutations in the Humsavar (green) and OMIM (red) sets for one amino acid mutating to all others. Each plot shows the results of mutation from a specific amino acid (e.g. Arg at top left) to every other amino acid.
(EPS)

Table S1 **The relative abundances of the various amino acids in the UniProt protein set.**
(PDF)
