# Supplementary material for: Amino Acid Changes in Disease-Associated Variants Differ Radically from Variants Observed in the 1000 Genomes Project Dataset
Source: PLoS Comput Biol. 2013 Dec 12;9(12):e1003382. doi: 10.1371/journal.pcbi.1003382 (PMC3861039; doi:10.1371/journal.pcbi.1003382)
Supplement: Table S1 — The relative abundances of the various amino acids in the UniProt protein set. (PDF) [file pcbi.1003382.s006.pdf]

Table S1. The relative abundances of the various amino acids in the UniProt protein set.

Amino acid	Abundance (%)
R	5.63083133373
K	5.79714973543
D	4.76732370533
E	7.13437004049
N	3.6117363067
Q	4.76916720684
S	8.30266991827
G	6.54206836837
H	2.63812747102
T	5.29415226919
A	6.99852702309
P	6.23374274109
Y	2.66393649214
V	5.92298791652
M	2.15610943601
C	2.2940936036
L	9.99640613222
F	3.69139093437
I	4.35942979346
W	1.19577957214
